# Comparative Analysis of Metabolites of Wild and Cultivated *Notopterygium incisum* from Different Origins and Evaluation of Their Anti-Inflammatory Activity

**DOI:** 10.3390/molecules30030468

**Published:** 2025-01-22

**Authors:** Fukang Kong, Yannan Kou, Xu Zhang, Yue Tian, Bin Yang, Weihao Wang

**Affiliations:** 1 State Key Laboratory for Quality Ensurance and Sustainable Use of Dao-di Herbs, Institute of Chinese Materia Medica, China Academy of Chinese Medical Sciences, Beijing 100700, China; fukangkong@outlook.com (F.K.); lidong8880@outlook.com (Y.K.); tulver1999@outlook.com (X.Z.); 2 School of Biomedicine, Beijing City University, No. 6 Huanghoudian Road, Haidian District, Beijing 100094, China; yuetian1201@outlook.com

**Keywords:** *Notopterygium incisum*, phytometabolomic, UHPLC-Orbitrap MS, GC–MS, coumarin, phenolic acid, anti-inflammation

## Abstract

The dried rhizome of *Notopterygium incisum* (NI) from the *Umbelliferae* family, genuinely produced in Sichuan, China, is a classic traditional Chinese medicinal herb for treating wind-dampness arthralgia. Due to scarce natural resources, wild NI is gradually being replaced by cultivated types. However, knowledge is limited regarding the differences in chemical composition and pharmacological effects between wild and cultivated NI and between Sichuan-grown and other-region-grown NI. In this study, a plant metabolomics strategy, based on GC–MS and UHPLC-Orbitrap MS, was employed to compare metabolic profiles between wild and cultivated NI and between cultivated NI from Sichuan and cultivated NI from Gansu and Qinghai. In total, 195 metabolites were identified, and the biosynthetic pathways of coumarins and phenolic acids, which were the most abundant secondary metabolites in NI, were summarized. Additionally, seven key metabolic intermediates were uncovered, revealing the reasons for the differences in metabolic profiles between wild and cultivated NI. The anti-inflammatory effect of wild and cultivated NI was verified by inflammatory gene expression and neutrophil count using a zebrafish yolk sac inflammation model. Overall, this study presents information on the types and synthesis of pharmacodynamic substances in NI and provides a basis for its cultivation and applications.

## 1. Introduction

*Notopterygium incisum* Ting ex H. T. Chang (NI) is a medicinal plant of the genus *Notopterygium* in the *Umbelliferae* family. This plant is mainly distributed in the provinces of Sichuan, Gansu, and Qinghai in China. The Aba Tibetan and Qiang Autonomous Prefecture areas in Sichuan Province are considered to be the origin of genuine NI production. The dried rhizome of NI is used clinically for the treatment of rheumatism and paralysis in traditional Chinese medicine [1]. Pharmacological studies have shown that NI extracts exhibit anti-inflammatory [2], antioxidant [3], antibacterial [4], analgesic [5], and anticancer [6] activities, as well as anti-osteoporosis [7] and neuroprotective [8] properties. Extracts of NI have also been used to treat Alzheimer’s disease [9]. Because of the scarcity of wild NI resources, it was ranked as a Grade III protected plant by the State Council of the People’s Republic of China in 1987 [10]. Consequently, artificial cultivated varieties have been developed, and currently, cultivated NI is the main source of this medicine provided in the market.

Phytochemical studies have shown that the chemical constituents in NI include volatile oils [11], phenolic acids, coumarins, polyene–alkynes and small amounts of flavonoids [12]. These secondary metabolites are unique substances produced by plants in response to environmental stress during growth. They allow plants adapt and survive in the environment, and are also the basis for the pharmacological effects of plant-derived drugs. Pharmacological studies have shown that phenolic acids and coumarins exhibit anti-inflammatory, analgesic, and antioxidant pharmacological activity [13,14]. Pharmacological studies on polyene–alkynes have demonstrated their anti-cancer properties and their ability to reduce neuroinflammation [15,16]. Although NI is a medicinal plant, it is still unknown whether the growth environment and cultivation practices affect the chemical composition of its secondary metabolites. In addition, whether changes in its chemical composition affect its therapeutic effects is also unknown. To date, few studies have compared the chemical composition and pharmacological activities between wild and cultivated NI or among cultivated NI from different growing areas.

In this study, we analyzed dried rhizomes of nine batches of wild and cultivated NI from Sichuan, Gansu, and Qinghai provinces. These samples were subjected to phytometabolomic analyses using gas chromatography–mass spectrometry (GC–MS) and ultrahigh performance liquid chromatography (UHPLC)-Orbitrap MS. This allowed us to compare metabolite profiles among wild resources, cultivars from genuine production areas, and cultivars from other origins. The biosynthetic pathway of phenolic acids and coumarins was summarized, and seven key intermediates contributing to differences in metabolic profiles among the samples were screened out. The possible mechanisms leading to differences in chemical profiles among the three types of NI resources were analyzed. Finally, the anti-inflammatory effects of wild NI and cultivated NI were compared using a zebrafish yolk sac inflammation model.

## 2. Results

### 2.1. Morphological Differences Among NI from Different Areas

**Figure 1:**
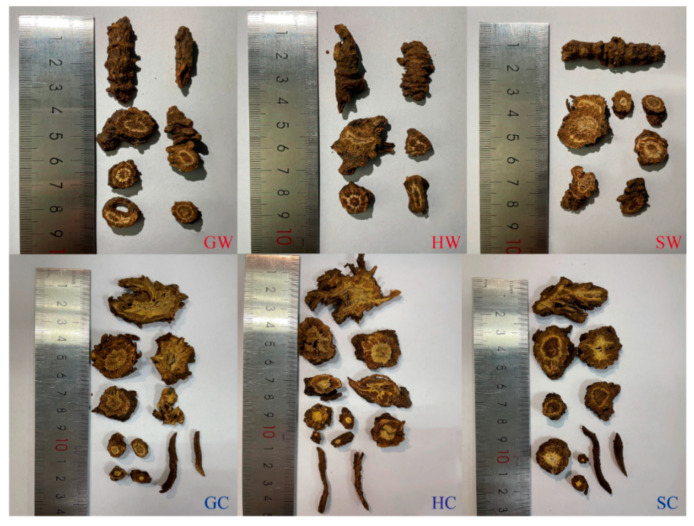
Morphological features of wild and cultivated *N. incisum* originating from different places. G: Gansu Province; H: Qinghai Province; S: Sichuan Province; W: wild; C: cultivated.

As shown in Figure 1, the morphology of rhizome slices of wild NI from Gansu, Qinghai, and Sichuan was similar. The rhizomes exhibited a brown surface, with punctate or verrucose protruding root scars at the nodes. The rhizome tissue displayed a radial arrangement, with deep fissures. The cortex was brown, the xylem was yellowish-white, and the pith was yellowish-brown. Cultivated NI exhibited a brown surface, a yellow-brown cortex, a yellow-white xylem, and many fibrous roots. The rhizome slices (“drinking tablets”) of the cultivated NI were generally larger than those of the wild NI. The rhizome slices of NI cultivated in Gansu and Qinghai were similar in their morphology, with larger fissures in the cross-section than those of NI cultivated in Sichuan. Their pith was yellow to light brown, whereas that of NI cultivated in Sichuan was brown.

### 2.2. Chemical Composition of Volatile Oil of NI as Determined by GC–MS

**Figure 2:**
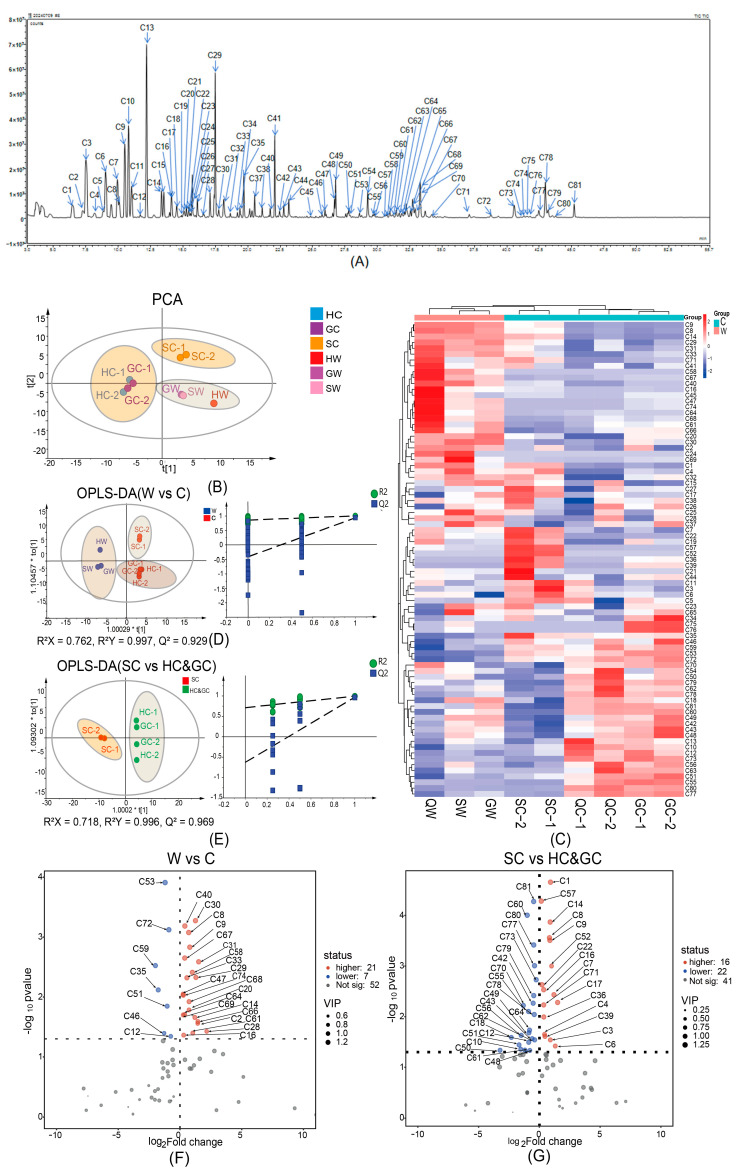
Multivariate statistical analyses of volatile components in nine NI samples (**A**) GC–MS (gas chromatography–mass spectrometry) chromatogram of wild NI from Sichuan Province. (**B**) PCA (principal component analysis) plot showing separation of samples based on their volatile component profiles. (**C**) Clustering heat map of NI samples based on their volatile component profiles. (**D**) OPLS-DA score plot and permutation test diagram of wild vs. cultivated NI based on their volatile component profiles. 1.0029*t[1] represents the scaling factor (1.0029) multiplied by t[1]; 1.10457*to[1] represents the scaling factor (1.10457) multiplied by to[1] (**E**) OPLS-DA score plots and permutation test plots of cultivated NI from Sichuan vs. cultivated NI from Qinghai and Gansu based on volatile oil components. 1.0002*t[1] represents the scaling factor (1.0002) multiplied by t[1]; 1.09302*to[1] represents the scaling factor (1.09302) multiplied by to[1] (**F**) Volcano plot showing differential volatile components between wild NI and cultivated NI. (**G**) Volcano plot showing differential volatile components between NI cultivated in Sichuan and NI cultivated in Qinghai and Gansu. G: Gansu Province; H: Qinghai Province; S: Sichuan Province; W: wild; C: cultivated.

The volatile components of the NI samples were analyzed by GC–MS. Figure 2A shows the total ion chromatogram (TIC) of wild NI from Sichuan (SW). Comparison of the data with those in the NIST database revealed a total of 81 chemical constituents (see Table S1 in Supplementary Material), including 33 monoterpenes, 33 sesquiterpenes, and 15 other components, with a total detection rate of ≥89.5%. The main volatile oil constituents included γ-terpinene (C13), (−)-4-terpineol (C29), bornyl acetate (C41), *p*-cymene (C10), (+)-4-carene (C9), and α-pinene (C3). Pharmacological studies have shown that γ-terpinene and α-pinene exhibit anti-inflammatory, antioxidant, neuroprotective, and analgesic effects [17,18,19]; *p*-cymene and (+)-4-carene have antibacterial, insecticidal, and antiviral effects [20,21,22]; (−)-4-terpineol shows anticancer effects [23]; and bornyl acetate displays anti-inflammatory and immunomodulatory effects [24].

A principal component analysis (PCA) was conducted for the batches of NI from various origins, using the relative abundance of chemical components as the variable (Figure 2B). The samples showed a clear clustering pattern in the PCA plot. The nine batches of samples were clustered into three categories: one category consisted of three batches of wild NI (from Sichuan, Gansu, and Qinghai), another consisted of four batches of cultivated NI from Qinghai and Gansu provinces, and the final category consisted of two batches of cultivated NI from Sichuan Province.

A cluster heat map was constructed to show the distribution of volatile components and their abundance in different batches of NI. As shown in Figure 2C, the distribution of volatile components was similar in the three wild NI samples from Sichuan, Gansu, and Qinghai. The distribution of volatile components was similar in cultivated NI from Qinghai and Gansu, but their volatile profiles differed from that of cultivated NI from Sichuan. The volatile profiles were similar in the two batches of cultivated NI from each place of origin. Thus, there was a high degree of inter-batch similarity in the volatile component composition of cultivated NI from the same planting area.

To identify the differentially accumulated metabolites (DAMs) between wild and cultivated NI, orthogonal partial least squares discriminant analysis (OPLS-DA) was performed to separate wild and cultivated NI from different origins on the basis of the relative contents of volatile components. The parameters to screen for DAMs between the wild and cultivated NI materials were a variable importance in projection (VIP) value of >1, Log_2_ fold change (FC) > 0 or <0, and *p* < 0.05. The OPLS-DA score plot and the permutation test results are shown in Figure 2D. The contents of 21 volatile components were higher in wild NI than in cultivated NI (11 monoterpenes, nine sesquiterpenes and one other compound). The contents of seven compounds were lower in wild NI than in cultivated NI (one monoterpene, four sesquiterpenes, and two other compounds).

The same procedure was used to identify DAMs between cultivated NI from Sichuan and cultivated NI from Gansu and Qinghai. The OPLS-DA score plot and permutation test results are shown in Figure 2E. The contents of 16 volatile compounds were higher in cultivated NI from Sichuan than in cultivated NI from Gansu and Qinghai (seven monoterpenes, three sesquiterpenes, and six other components). The contents of 22 compounds were lower in cultivated NI from Sichuan than in cultivated NI from Gansu and Qinghai (five monoterpenes, 14 sesquiterpenes, and three other compounds).

Volcano plots were constructed to visualize the differential composition of volatile compounds in wild NI vs. cultivated NI and in cultivated NI from Sichuan vs. cultivated NI from Gansu and Qinghai (Figure 2F,G). As shown in Figure 2F, the contents of α-phellandrene (C8), (+)-4-carene (C9), α-terpineol (C31), γ-muurolene (C58), copaene (C47), and dehydroxy-isocalamendiol (C74) were higher in wild NI than in cultivated NI, whereas the contents of cis-thujopsene (C53), guaiac alcohol (C72), β-chamigrene (C59), caryophyllene (C51), and E-7-tetradecenol (C46) were higher in cultivated NI than in wild NI. Pharmacological studies have shown that α-phellandrene and copaene have anti-inflammatory and analgesic pharmacological effects [25,26]; α-phellandrene also exhibits antioxidant and wound-healing promoting effects [27]; caryophyllene displays anti-inflammatory and antioxidant effects [28]. These results show that, compared with cultivated NI, wild NI contained more volatile components that exhibit antioxidant and anti-inflammatory pharmacological activities.

Next, we compared the volatile profiles between cultivated NI from Sichuan and cultivated NI from Gansu and Qinghai. The contents of α-Terpinolene (C14), (+)-4-carene (C9), cubenene (C52), octanal (C7), and nonanal (C17) were higher in cultivated NI from Sichuan than in cultivated NI from Gansu and Qinghai, whereas the contents of α-bisabolol (C81), (−)-aristolene (C77), apiol (C73), and guaiol (C78) were higher in cultivated NI from Gansu and Qinghai than in cultivated NI from Sichuan. Octanal and nonanal have bacteriostatic effects [29,30]; (+)-4-carene, α-bisabolol, and guaiol show insecticidal and bacteriostatic effects [21,22,31,32]; α-bisabolol also exhibits anticancer and anti-inflammatory pharmacological effects [33,34]. In summary, NIs cultivated in Sichuan, Gansu, and Qinghai were rich in volatile compounds with a range of antibacterial and insecticidal effects.

### 2.3. Non-Volatile Component Profiles of NI Samples as Determined by UHPLC-Orbitrap MS Analysis

**Figure 3:**
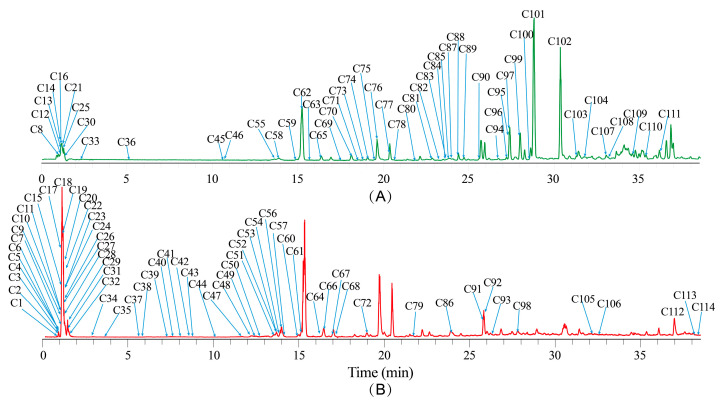
TIC (total ion chromatogram) of QC sample acquired by UHPLC-Orbitrap MS. (**A**) TIC in positive ion mode; (**B**) TIC in negative ion mode.

The chemical component of the 95% ethanol extract of nine batches of samples was investigated by UHPLC-Orbitrap MS. Figure 3 shows the TICs of the QC sample in both positive and negative ion modes. A total of 114 compounds, including secondary metabolites and endogenous substances, were identified (see Table S2), consisting of 51 coumarins, 19 phenolic acids and their derivatives, two flavonoids, three polyene–alkynes, 14 amino acids, four nucleosides, six carbohydrates, seven fatty acids, five amides, and three other compounds. The chemical structures of these compounds are shown in Supplementary Figure S1. As shown in Figure 3, the intensities of the peaks corresponding to the coumarin constituents such as nodakenin (C62), nodakenitin (C76), angelicin (C77), imperatorin (C95), notopterol (C97), phellopterin (C99), and isoimperatorin (C101), as well as the intensity of the peak corresponding to falcarindiol (C102), were significantly higher than those of the other constituents.

**Figure 4:**
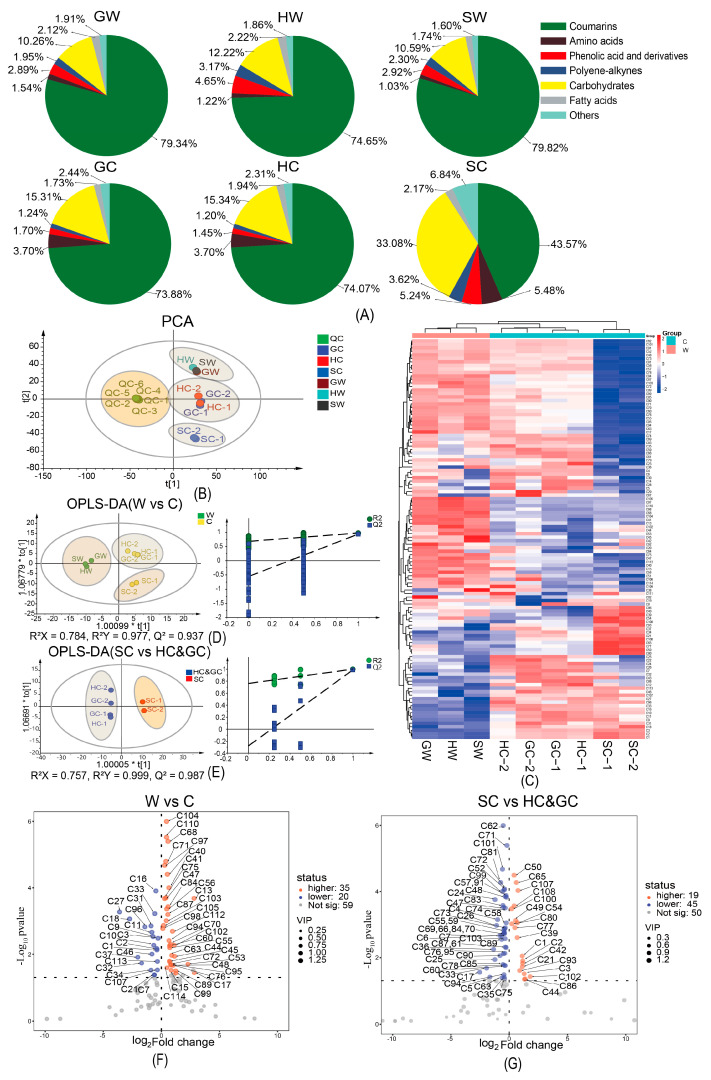
Multivariate statistical analysis of non-volatile metabolites in different batches of NI. (**A**) Sector chart of the proportion of various components in wild and cultivated NIs from different growing areas. (**B**) PCA score diagram of samples. (**C**) Clustering heat map based on contents of constituents in nine batches of NI samples. (**D**) OPLS-DA score diagram and permutation test result for wild NI vs. cultivated NI. (**E**) OPLS-DA score diagram and permutation test result for cultivated NI from Sichuan vs. cultivated NI from Qinghai and Gansu. (**F**) Volcano plot of DAMs between wild and cultivated NI. (**G**) Volcano diagram of DAMs between cultivated NI from Sichuan and cultivated NI from Qinghai and Gansu. G: Gansu Province; H: Qinghai Province; S: Sichuan Province; W: wild; C: cultivated.

The identified chemical components were classified into seven categories: coumarins, phenolic acids, polyene–alkynes, amino acids, carbohydrates, fatty acids, and other constituents (including flavonoids, nucleosides, amides, and others). Based on the sum of peak areas of each category of constituents, sector charts were constructed to visualize the distribution of these categories in the different samples (Figure 4A). As shown in the figure, coumarins were the dominant secondary metabolites in NI, followed by phenolic acids. The mass percentage of phenolic acids was higher in wild NI than in cultivated NI from Gansu and Qinghai, but lower in wild NI from Sichuan than in cultivated NI from Sichuan.

Polyene–alkynes exhibit anti-cancer properties and reduce neuroinflammation [15,16]. The mass percentage of polyacetylenes was higher in cultivated NI from Sichuan than in wild NI from Sichuan. The mass percentages of amino acids and carbohydrates were higher in cultivated NI than in wild NI. The mass percentage of coumarins was significantly lower in cultivated NI from Sichuan than in wild NI from Sichuan.

Using the peak areas of the identified components as the variable, PCA clustering analysis was performed. As shown in the PCA plot (Figure 4B), the QC samples were clustered together, indicating good instrument precision and reliable mass spectrometry data results. In the plot, wild NI from Sichuan, Gansu, and Qinghai were grouped together; NI cultivated in Qinghai and Gansu were clustered together; and NI cultivated in Sichuan was placed in a separate category. We further investigated the distribution of secondary metabolites in different batches of NI samples using heat maps (Figure 4C). Wild NI from the three different growing areas exhibited similar color block distributions in the heat map. The color distribution was similar for NI cultivated in Gansu and Qinghai, while that of NI cultivated in Sichuan was significantly different from the other samples.

To clarify the DAMs between wild and cultivated NI, as well as between NI cultivated in Sichuan and NI cultivated in Gansu and Qinghai, OPLS-DA analysis was first executed using the peak areas of non-volatile components in wild and cultivated NI as the variable. The OPLS-DA score diagram and permutation test results are shown in Figure 4D. Using VIP > 1, Log_2_FC > 0 or <0, and *p* < 0.05 as screening criteria, the DAMs between wild and cultivated NI were screened. The contents of 35 components were higher in wild NI than in cultivated NI, including 20 coumarins (eight simple coumarins, 11 linear furancoumarins, and one angular furancoumarin), three polyene–alkynes, two carbohydrates, six phenolic acids and their derivatives, two fatty acids, one amide, and one other component. The contents of 20 components were lower in wild NI than in cultivated NI, including three coumarins (two simple coumarins, one linear furancoumarin), seven amino acids, one nucleoside, two carbohydrate component, two phenolic acids and their derivatives, two fatty acids, two amides, and one other chemical component.

Using the same method, we identified 64 DAMs between NI cultivated in Sichuan and NI cultivated in Qinghai and Gansu. The OPLS-DA score plot and permutation test results are shown in Figure 4E. The contents of 19 compounds were higher in NI cultivated in Sichuan than in NI cultivated in Qinghai and Gansu, including seven coumarin components (four simple coumarins, two linear furancoumarins, and one angular furancoumarin), three amino acids, one nucleoside, one flavonoid, one polyene–alkyne, and six phenolic acids and their derivatives. The contents of 45 compounds were lower in NI cultivated in Sichuan than in NI cultivated in Qinghai and Gansu, including 33 coumarins (seven simple coumarins, 20 linear furancoumarins, six angular furancoumarins), four amino acids, three nucleosides, one carbohydrate, three phenolic acids, and one other chemical component.

Volcano plots were constructed to visualize the DAMs between wild and cultivated products, as well as between cultivated NI from Sichuan and Gansu and Qinghai (Figure 4F,G). As shown in Figure 4F, there were higher contents of falcarinol (C104), azelaic acid (C68), notopterol (C97), aesculatin (C40), caffeic acid (C41), osthenol (C87), marmesin (C70), falcarindol (C102), and ferulic acid (C53) in wild NI than in cultivated NI. In contrast, the contents of proline (C16), phenyalanine (C33), asparagine (C11), D-sucrose (C18), succinic acid (C27), and cinnamic acid (C37) were higher in cultivated NI than in wild NI. This phenomenon, where substances related to the tricarboxylic acid (TCA) cycle, such as aspartic acid and succinic acid, were more abundant in cultivated NI than in wild NI indicates that cultivated NI, directed more resources to development and less to secondary metabolism.

Next, we compared the cultivated NI from Sichuan with cultivated NI from Gansu and Qinghai. The contents of umbelliferone (C50), diosmin (C65), chlorogenic acid (C39), *p*-coumaroyl quinic acid (C44), ornithine (C1), arginine (C2), histidine (C3), and falcarindol (C102), were higher in cultivated NI from Sichuan than in cultivated NI from Gansu and Qinghai. In contrast, the contents of nodakenin (C62), isomperatin (C101), bergapten (C81), bergaptol (C72), *p*-coumaric acid (C47), D-fructopyranose (C17), and phenyalanine (C33) were higher in cultivated NI from Gansu and Qinghai than in cultivated NI from Sichuan.

### 2.4. Pathway Enrichment and Metabolic Pathway Analysis of DAMs

**Figure 5:**
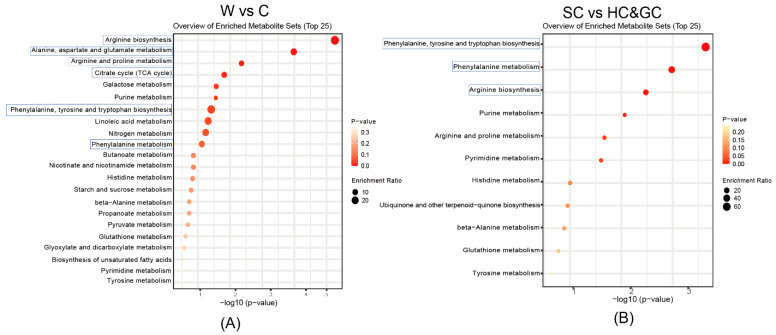
Pathway enrichment analysis of DAMs. (**A**) Enrichment analysis of metabolic pathways enriched with DAMs in W vs. C. (**B**) Enrichment analysis of metabolic pathways enriched with DAMs in SC vs. HC and GC.

To further reveal the molecular biological mechanisms leading to differences in chemical composition among the NI samples, enrichment analysis was conducted to identify the metabolic pathways enriched with DAMs using the Kyoto Encyclopedia of Genes and Genomes (KEGG) and MetaboAnalyst. The DAMs between wild and cultivated NI were enriched in 22 metabolic pathways (Figure 5A), including arginine biosynthesis (map00220); alanine, aspartate, and glutamate metabolism (map00250); arginine and proline metabolism (map00330); the TCA cycle (map00020); and phenylalanine metabolism (map00360). The DAMs between NI cultivated in Sichuan and NI cultivated in Qinghai and Gansu (Figure 5B) were enriched in 11 pathways, including phenylalanine, tyrosine and tryptophan biosynthesis (map00400); phenylalanine metabolism; and arginine biosynthesis.

Phenolic acids and coumarins are important therapeutic components of NI. These compounds display various pharmacological properties, such as anti-inflammatory, analgesic, and antioxidant activities [13,14]. Phenylalanine (C00079) is a substrate for the biosynthesis of phenolic acids and coumarins. First, it is converted into *p*-coumaric acid (C00811) by L-phenylalanine ammonia-lyase (PAL) and cinnamic acid-4-hydroxylase, and then *p*-coumaric acid is converted into *p*-coumaric acid CoA by 4-coumarite: coenzyme A ligase. *p*-Coumaric acid CoA plays several roles—it is used in the synthesis of phenolic acids (caffeic acid (C01481), ferulic acid (C01494), etc.) through the shikimic acid pathway or in the synthesis of chlorogenic acid (C00852) through the action of coumaric acid 3′- hydroxylase (C3′H) [35,36]. *p*-Coumaric acid CoA, via dihydroxycinnamoyl CoA, is also used in the synthesis of umbelliferone (C09315), which gives rise to a series of coumarins with complex structures under the action of C-prenyltransferase (C-PT) and cyclases.

**Figure 6:**
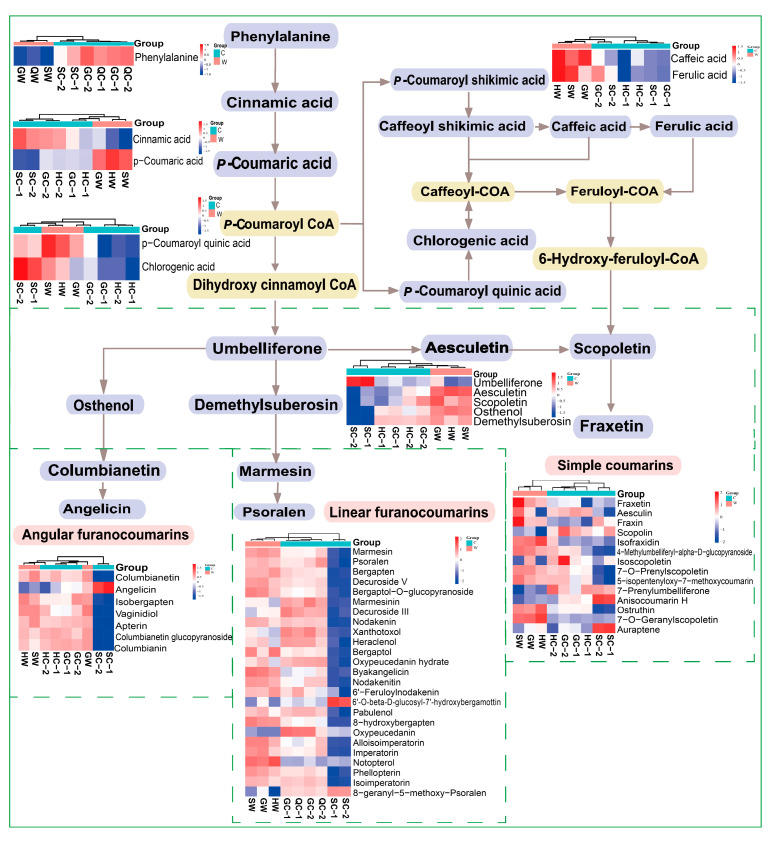
Phenolic acid and coumarin biosynthesis pathways construct in nine batches of NI. G: Gansu Province; H: Qinghai Province; S: Sichuan Province; W: wild; C: cultivated.

**Figure 7:**
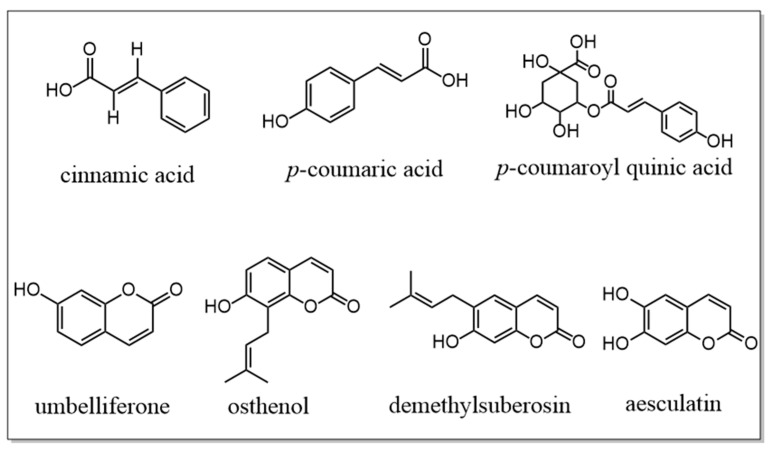
Chemical structure of seven important intermediates.

The biosynthetic pathways of the phenolic acids and coumarins in NI are shown in Figure 6. In the pathway where phenolic acids and coumarins are synthesized from phenylalanine, there are seven important intermediates that affect the levels of phenolic acids/coumarins, namely cinnamic acid, *p*-coumaric acid, *p*-coumaroyl quinic acid, umbelliferone, osthenol, demethylsuberosin, and aesculatin. Their structural formulas are shown in Figure 7. The *p*-coumaric acid contents were higher in wild NI than in cultivated NI, so the corresponding downstream products caffeic acid and ferulic acid also had relatively high contents in wild NI. In addition, the *p*-coumaroyl quinic acid content was slightly lower in NI cultivated in Sichuan cultivated than in wild NI, but the chlorogenic acid content was significantly higher in NI cultivated in Sichuan than in wild NI. We speculate that this may be due to the higher expression level of coumaroyl quinic acid 3′- hydroxylase in cultivated NI from Sichuan.

Coumarin synthase catalyzes the production of the structurally complex coumarin umbelliferone from dihydroxy cinnamoyl CoA, which is generated from *p*-coumaric acid CoA. Then, osthenol (C18080) is produced by the action of umbelliferone 6-phenyltransferase. The intramolecular cyclization of osthenol by osthenol cyclase forms angular furanocoumarins; alternatively, umbelliferone 8-prenyltransferase can generate demethylsuberosin (C18083). Then, the intramolecular cyclization of demethylsuberosin gives rise to linear furanocoumarins [37]. The umbelliferone content was significantly higher in NI cultivated in Sichuan than in the other samples, but the osthenol and demethylsuberosin contents were lower in NI cultivated in Sichuan than in the other samples. These two products are precursors for the synthesis of structurally diverse angular and linear furanocoumarins. Therefore, the total contents of angular furanocoumarins and linear furanocoumarins were lower in NI cultivated in Sichuan than in the other samples. In addition, the contents of simple coumarins, such as aesculatin (C09263), were lower in NI cultivated in Sichuan than in wild NI.

Overall, the coumarin content was lower in NI cultivated in Sichuan than in wild NI. For the same reason, although the umbelliferone content was not significantly higher in NI cultivated in Sichuan than this in wild NI, the contents of the precursor substances (osthenol and demethylsuberosin) of angular and linear furanocoumarins were significantly higher in the wild NI samples. Overall, therefore, the contents of angular and linear furanocoumarins were significantly higher in wild NI than in NI cultivated in Sichuan.

**Figure 8:**
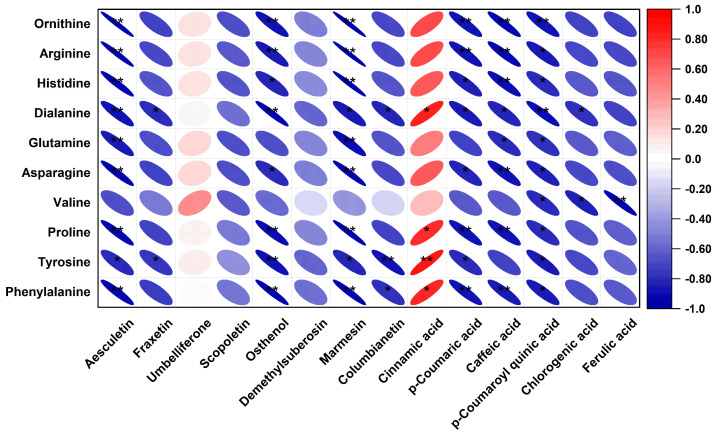
Correlation analysis between amino acids and key phenolic acid and coumarin components in NI. Asterisks indicate significance of correlation (* significant at *p* < 0.05; ** significant at *p* < 0.01). Oval in red color: positive correlation; oval in blue color: negative correlation.

To explore the relationships between endogenous metabolites and the biosynthesis of phenolic acids and coumarins, we conducted a correlation analysis between amino acids and key intermediates in the phenolic acid and coumarin biosynthetic pathways. The results show that, except for cinnamic acid and umbelliferone, all the other phenolic acids and coumarins were negatively correlated with amino acids (Figure 8). No previous studies have reported a direct impact of amino acids on coumarin biosynthesis. However, according to a study on the germination of *Eleusine indica* seeds, coumarins are allelochemical substances. Exposure of *E. indica* seeds to coumarins resulted in significant changes in their amino acid profile and significantly affected the expression of genes related to the TCA cycle [38]. In our study, asparagine and glycine, which are both related to the TCA cycle, and arginine and proline, which are related to nitrogen metabolism, showed significant negative correlations with some coumarins. These results suggest that there is a certain degree of competition between coumarin production and pathways related to development in NI.

According to its chemical structure, phenylalanine may be metabolized to generate cinnamic acid and tyrosine. Accordingly, in our correlation analysis, we detected a positive correlation between phenylalanine and cinnamic acid. This suggests that, to improve the yield of cinnamic acid and increase the contents of downstream phenolic acids and coumarins, specific enzymes could be up-/downregulated to minimize the amount of tyrosine generated from phenylalanine and increase the amount of cinnamic acid.

### 2.5. Results of Anti-Bacterial Inflammation Pharmacodynamic Study

#### 2.5.1. Evaluation of Anti-Inflammatory Effect of NI Treatment by Neutrophil Counts

**Figure 9:**
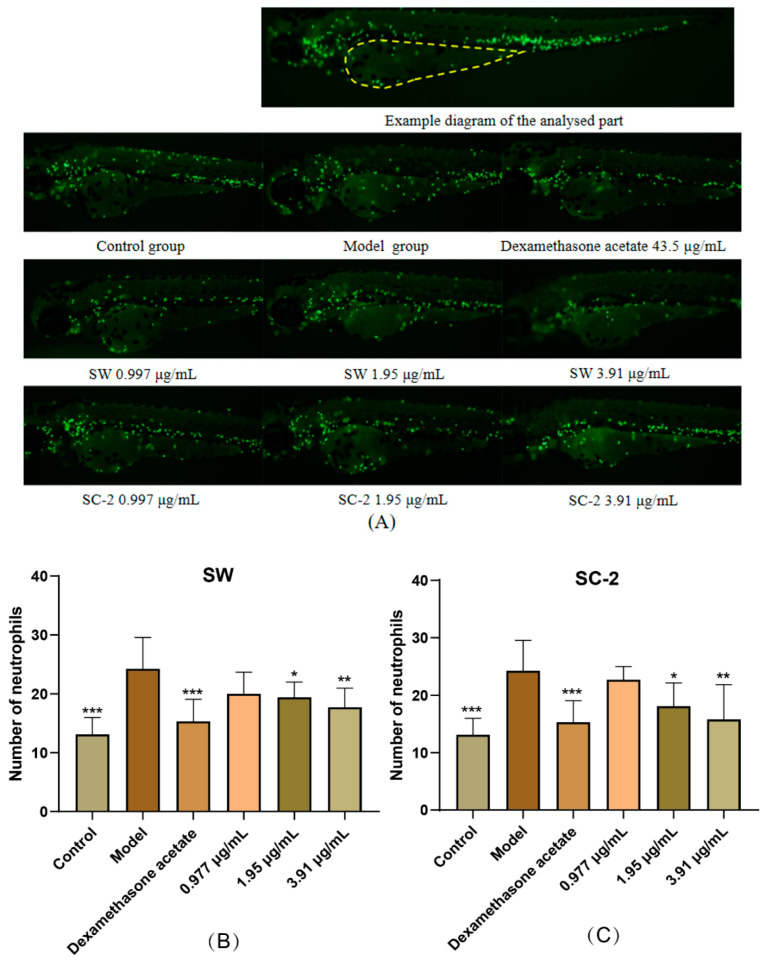
Neutrophil visualization and counts as indexes of anti-inflammatory effects of SW and SC-2 in the zebrafish yolk sac system. (**A**) Recruitment of neutrophils to zebrafish yolk sac in control, model, and positive control groups, as well as in groups treated with Sichuan wild NI (SW) and Sichuan cultivated NI (SC-2) at different doses. (**B**) Histogram of neutrophil counts in zebrafish yolk sac treated with different doses of SW. (**C**) Histogram of neutrophil counts in zebrafish yolk sac treated with different doses of SC-2. Yellow dashed area in (**A**) represents the location of zebrafish yolk sac analysis; green fluorescent particles represent neutrophils. Compared with model group, *, **, and *** indicate significant differences at *p* < 0.05, *p* < 0.01, and *p* < 0.001, respectively.

The neutrophil counts were significantly higher in the model group than in the control group (*p* < 0.001), indicating the successful establishment of the zebrafish inflammation model. Both SW and SC-2 exhibited anti-inflammatory effects at medium and high doses, as evidenced by significantly lower neutrophil counts than those observed in the model group. Images of the neutrophils and bar charts of the neutrophil counts in zebrafish yolk sacs are presented in Figure 9. The neutrophil count numbers are provided in Supplementary Material Table S3.

#### 2.5.2. Effect of NI Treatments on Transcript Levels of Genes Encoding Inflammation Markers

**Figure 10:**
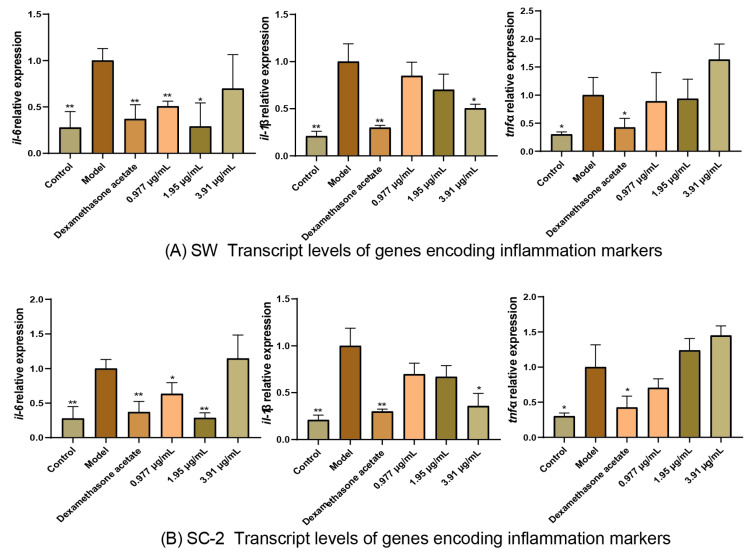
qRT-PCR results showing transcript levels of genes encoding inflammatory factors in the zebrafish inflammation model. (**A**) Histogram of transcript levels of genes encoding inflammation markers in zebrafish after SW administration. (**B**) Histogram of transcript levels of genes encoding inflammation markers in zebrafish after SC-2 administration. Compared with model group, * and ** indicate significant difference at *p* < 0.05 and *p* < 0.01, respectively.

To evaluate the anti-inflammatory pharmacodynamic effects of SW and SC-2, the transcript levels of *IL-1β*, *IL*-6, and *TNF-α* were determined by qRT-PCR. As shown in Figure 10, *IL-1β*, *IL-6*, and *TNF-α* were significantly upregulated in the model group compared with the control group, indicating successful establishment of the inflammation model. Compared with the model group, the groups treated with low and medium doses of SW and SC-2 showed the downregulation of *IL-6*, and those treated with high doses of SW and SC-2 showed the downregulation of *IL-1β*. The transcript level of *TNF-α* was not significantly affected by SW or SC-2 at any dose. In conclusion, both SW and SC-2 exerted anti-inflammatory effects, observed as decreases in the transcript levels of the inflammatory marker genes *IL-1β* and *IL-6*. The gene transcript level data are provided in Supplementary Material Table S4.

## 3. Materials

Material: Samples of *Notopterygium incisum* (NI) were collected from Sichuan, Qinghai, and Gansu provinces. These samples were identified as the dried rhizomes of *Notopterygium incisum* Ting ex H. T. Chang, a plant belonging to the *Umbelliferae* family, by Yang Bin, a researcher at the Institute of Traditional Chinese Medicine of the Chinese Academy of Chinese Medical Sciences. Voucher specimens have been deposited in the Institute of Traditional Chinese Medicine, Chinese Academy of Chinese Medical Sciences. Detailed sample information is presented in Table 1.

Animals: The zebrafish were kept in culture water at 28 °C (water quality: 200 mg instant sea salt per 1 L reverse osmosis water, with conductivity of 450–550 μS/cm, pH of 6.5–8.5, and hardness of 50–100 mg/L CaCO_3_). The fish were bred at the fish culture center of HuanTe Bio-Technology Co Ltd. The license number of the experimental animals was SYXK (Zhejiang) 2022-0004. The feeding and management practices complied with the requirements of the international AAALAC certification (certification number: 001458), IACUC ethics review number: IACUC-2024-9701-01.

Reagents: The reagents used in this study (and their manufacturers) were as follows: distilled water (Guangzhou Watsons Food and Beverage Co., Ltd., Guangzhou, China), ethyl acetate (AR, Xilong Science, Shantou, China), anhydrous sodium sulfate (AR, Shanghai McLean Biochemical Technology Co., Ltd., Shanghai, China), ethanol (AR, Tongguang Fine Chemicals company, Beijing, China), dimethyl sulfoxide (DMSO, Sigma, St Louis, MO, USA), methyl cellulose (Shanghai Aladdin Biochemical Technology Co., Ltd., Shanghai, China), and acetonitrile (GR, Thermo Fisher Scientific, Waltham, MA, USA).

Trial drugs: Lipopolysaccharide (LPS, Sigma, Saint Louis, MO, USA) and dexamethasone acetate (Shanghai Aladdin Biochemical Technology Co., Ltd., Shanghai, China) were used for the pharmacological experiments. Information regarding the reference standards used for chemical composition identification is provided in Supplementary Material Table S5.

## 4. Methods

### 4.1. Preparation of Samples for Chemical Components Analysis

Preparation of extracts for analysis of volatile components: The dried rhizome of NI was crushed and passed through a No. 3 sieve to obtain the sample powder. Then, 100.00 g of the sample powder was mixed with 600 mL water and a small amount of zeolite, allowed to stand for 9 h, then heated for 12 h. The upper phase was collected as the volatile oil sample. For each sample, 0.1 mL volatile oil was mixed with 0.4 mL ethyl acetate, and then anhydrous sodium sulfate was added to remove the water. The mixture was shaken well and kept at 4 °C overnight; then, an aliquot was used for the analysis of volatile compounds using GC–MS.

Preparation of extract for analysis of non-volatile components: A 0.2 g portion of each powdered NI sample (sieved through a No. 3 sieve) was weighed precisely, and then 20 mL of 95% *v/v* ethanol was added. The mixture was subjected to ultrasonic treatment for 30 min (KQ-250DB, Kunshan Ultrasonic Instrument Co., Ltd., Suzhou, China) and then centrifuged for 10 min (9391× *g*). A portion of the supernatant was filtered (0.22 μm cellulose membrane filter) before analysis of the non-volatile components by UHPLC-Orbitrap MS.

QC test solution preparation: Samples of the nine batches of *N. incisum* powder (sieved through a No. 3 sieve) were combined to create a QC sample. To generate the mixed sample for QC, 22.30 mg of each sample was weighed precisely into a conical flask, and then 20 mL of 95% ethanol was added. The mixture was subjected to ultrasonic extraction for 30 min and then centrifuged for 10 min (9391× *g*). The supernatant was filtered through a cellulose membrane filter (0.22 μm) before analysis.

### 4.2. Preparation of Samples for Analysis of Anti-Inflammatory Activity

Two samples were used in the anti-inflammatory experiment, i.e., wild NI from Sichuan and cultivated NI from Sichuan. For each sample, 4 g of powder (sieved through a No. 3 sieve) was weighed into a 50 mL triangular flask. The sample was extracted with 25 mL of 95% *v/v* ethanol three times, for 30 min each, and the three extracts were combined. The extracts were concentrated under reduced pressure to remove the ethanol, and then freeze-dried (Alpha 2–4, LSCbasic laboratory freeze-dryer, Martin Christ, Osterode am Harz, Germany) to obtain the extract powder for the anti-inflammatory pharmacodynamic experiments.

### 4.3. Determination of the Chemical Components of NI Volatile Oil by GC–MS

GC conditions: The gas chromatograph (Thermoscientific TRACE 1600, Thermo Fisher Scientific, Waltham, MA, USA) was equipped with a Thermoscientific TG-5SILMS (0.25 μm, 0.25 mm × 30 m) chromatographic column (Thermo Fisher Scientific, Waltham, MA, USA). The operating conditions were as follows: carrier gas, helium; inlet temperature, 250 °C; detector temperature, 250 °C; sample volume, 2 µL. The programmed heating conditions were as follows: a starting temperature of 50 °C, increasing to 130 °C at a rate of 3 °C·min^−1^, then to 137 °C at a rate of 0.5 °C·min^−1^, and then to 180 °C at a rate of 4.3 °C·min^−1^, and held at 180 °C for 5 min.

Mass spectrometry conditions: The mass spectrometer (Thermoscientific TSQ 9610, Thermo Fisher Scientific, Waltham, MA, USA) was operated with an EI ion source, with electron energy of 70 eV; a scanning interval of 0.30 s; a mass scanning range of 35–500 Da; and a gas flow rate of 1 mL·min^−1^.

### 4.4. Determination of Chemical Components of NI by UHPLC-Orbitrap MS

UHPLC conditions: The ultra-high performance liquid chromatograph (Thermo Scientific Vanquish) was equipped with a MORHCHEM Caprisil C18-X (1.8 μm, 100 mm × 2.1 mm) column (Morhchem, City of Industry, CA, USA). The samples were eluted by gradient elution with the mobile phase consisting of high purity water containing 0.01% acetic acid (solvent A) and acetonitrile (solvent B). The elution program was as follows: 0–9 min, 5–12% B; 9.00–18.00 min, 12–31% B; 18–36 min, 31–90% B; 36–36.5 min, 90–98% B; 36.5–38.5 min, 98% B; 38.6–43 min, 5% B. The column temperature was 35 °C, and the injection volume was 2 µL.

Mass spectrometry conditions: The mass spectrometer (Thermo Orbitrap Exploris 120, Thermo Fisher Scientific, Waltham, MA, USA) was operated with an electrospray ionization source. The data were collected in positive and negative ion modes, respectively. The operating conditions were as follows: positive ion spray voltage, 3.50 kV; negative ion spray voltage, −3.00 kV; sheath gas, 40 arb; auxiliary gas, 10 arb. The temperature of the ion transfer tube was 320 °C, and a primary full scan was performed at a resolution of 120,000, with a primary ion scan range of 100–1500 *m/z*. Secondary cleavage was performed using an HCD, with a collision energy parameter set at 30% and a secondary resolution of 30,000. The ions in the first four collected signals were fragmented, and dynamic exclusion was used to remove interfering signals.

### 4.5. Anti-Inflammatory Pharmacodynamic Experiments

A pharmacological experiment was conducted in which zebrafish were used as the experimental animals, and a bacterial inflammation model was established by injecting lipopolysaccharide (LPS) into the yolk sac.

Cytokines such as *IL-6*, *IL-1β*, and *TNF-α* are inflammatory markers, and their levels are commonly used indicators for evaluating the inflammatory effect of a drug or compound [39]. The determination of transcript levels of cytokine-related genes as indexes of the inflammatory response can eliminate the interference of multiple factors in the body’s response. Compared with measurements of these cytokines, measurements of their gene transcript levels are a better index of inflammation because the gene expression response is faster and more sensitive, as well as more informative, in revealing the mechanism of drug action.

To evaluate the pharmacological effects of oral administration of 95% ethanol extracts of wild and cultivated NI from Sichuan (SW and SC-2, respectively) on bacterial inflammation, we conducted quantitative real-time polymerase chain reaction (qRT-PCR) analyses to determine the transcript levels of *IL-6*, *IL-1β*, and *TNF-*α and compared neutrophil counts among the control, experimental, model control, and positive control groups.

#### 4.5.1. Maximum Detectable Concentration Determination

Transgenic neutrophil green fluorescent zebrafish (MPX) were randomly selected 3 days post-fertilization (3 dpf) and added to 6-well plates (Zhejiang Bellambeau Biotechnology Co. Ltd., Hangzhou, China), with 30 tails per well. The NI samples were applied as an aqueous solution, and the treatment, model, and control groups were established with a volume of 3 mL per well. After 1 h of sample pretreatment, the yolk sacs of all experimental groups (except the normal control group) were injected with LPS using a microinjector (IM300, Narishige, Tokyo, Japan) to establish a bacterial inflammation model. The minimum detectable concentrations (MTCs) of the samples in the modeled zebrafish were determined after treatment at 28 °C for 2 h. The results are shown in Supplementary Material Table S6.

#### 4.5.2. Effects of NI Samples on Bacterial Inflammation (Neutrophil Counts)

As described above, 3 dpf MPX were randomly selected and added to 6-well plates, with 30 tails per well. The NI samples were applied in the form of aqueous extracts, and the positive control was dexamethasone acetate with a concentration of 43.5 μg/mL. The control, model, and treatment groups were established with a total volume of 3 mL per well. After 1 h of sample pretreatment, the yolk sacs of each experimental group (except the normal control group) were injected with LPS to establish the inflammation model. After 2 h of treatment at 28 °C, 10 zebrafish tails were randomly selected from each experimental group and observed and photographed under a fluorescence microscope (AZ100, Nikon, Tokyo, Japan). The images were analyzed and processed using NIS-Elements D 3.20 advanced image processing software. This allowed us to count the number of neutrophils in the zebrafish yolk sac.

#### 4.5.3. Effect of NI Samples on Gene Expression in the Zebrafish Inflammation Model

The normal control, model, and treatment groups were established with 3 dpf MPX (30 tails per well), as described in Section 4.5.2. After 1 h of sample pretreatment, the yolk sac of each experimental group (except the normal control group) was injected with LPS to establish the inflammation model. After 2 h of treatment at 28 °C, RNA was extracted from the MPX in each group using an RNA Rapid Extraction Kit (TL2204001643C, Foshan Aowei Biotechnology Co., Ltd., Foshan, China). The concentration and purity of the total RNA were determined using a UV–visible spectrophotometer (Nanodrop 2000, Thermo Fisher Scientific, Waltham, MA, USA). The results are shown in Supplementary Material Table S7. The transcript levels of the genes encoding inflammatory factors were determined by qRT-PCR, using the primer sequences shown in Table S8.

For the qRT-PCR analyses, 2.00 μg of total RNA from each sample was used to synthesize cDNA in a 20.0-μL reaction mixture, using a cDNA First Strand Synthesis Kit (X0320, Tiangen Biochemical Science and Technology Co., Ltd., Beijing, China). The transcript levels of *β-actin*, *IL-1β*, *IL-6*, and *TNF-α* were detected by qRT-PCR (T100, Bio-Rad, Hercules, CA, USA). The relative transcript levels of *IL-1β*, *IL-6*, and *TNF-α* were calculated using *β-actin* as an internal reference.

### 4.6. Data Analysis

#### 4.6.1. Methods for Identification of Chemical Components

Volatile components analysis: Qualitative and semi-quantitative analyses of the volatile oil composition of the nine batches of samples were carried out by comparing the data obtained in our GC–MS analyses with those in the NIST database. The NIST database was searched, and the chemical constituents of the volatile oils were identified based on a match rate greater than 800. The chromatographic peaks of the nine batches of volatile samples were integrated, and the content of each volatile oil component was expressed as the relative peak area (%).

Non-volatile components analysis: The raw mass spectral data acquired by UHPLC-Orbitrap MS were imported into Compounds Discoverer 3.3 software (Thermo Scientific, USA) connected with the online KEGG (https://www.kegg.jp/, accessed on 12 July 2024), ChEBI (https://www.ebi.ac.uk/chebi/, accessed on 12 July 2024), ChEMBL (https://www.ebi.ac.uk/chembl/, accessed on 12 July 2024), mzCloud (https://www.mzcloud.org/, accessed on 12 July 2024), and in-house Thermo Scientific mzVault databases, as well as other libraries. After peak filtering, peak alignment, and peak identification, a data matrix containing information such as retention time (RT), *m/z*, compound name, peak area, etc., was generated. Then, all identified compounds in this matrix were confirmed either by referring to the reference standard compound information regarding RT and *m/z* or by analyzing the first and second stage *m/z* information, based on the compound’s mass spectrometric fragmentation regularity. In the qualitative analysis of the metabolites, interference from the isotopic signals, duplicate signals from the K^+^ and NH_4_^+^ ions, and fragment ions from other larger molecules were removed. The deviation was set to <5 × 10^−6^. The content of each chemical component in the sample is represented by the peak area.

#### 4.6.2. Phytometabolomic Research Based on Multivariate Statistical Analysis

The qualitative and semi-quantitative results of the chemical components of the samples underwent multivariate statistical analyses, including cluster analysis (CA), PCA, and OPLS-DA. The CA grouped samples according to the distribution of metabolites, and the heatmap was drawn online using SRplot (https://www.bioinformatics.com.cn, accessed on 27 September 2024). The clustering approach is designated as “complete”, the distance measure is opted for “Euclidean”, and the callback function is set to “pheatmap”.

The PCA clustered the samples on the basis of similarities in the volatile oil components and the 95% ethanol extract components, including quality control samples (QC), using SIMCA 14.1 software (Umetrics, Malmö , Sweden). The scaling type was set as unit variance (UV). For the PCA of the volatile oil components, the cumulative contribution rate of five principal components reached 0.935, and the value of Q^2^ was 0.59. Meanwhile, for the PCA of the 95% ethanol extract components, the cumulative contribution rate of three principal components reached 0.846, and the value of Q^2^ was 0.572, indicating that the models possess good predictive ability.

Then, OPLS-DA was performed to calculate the VIP value for the screening of DAMs. The validity of the OPLS-DA model was assessed using the permutations function. The predictive parameters for evaluating the model included R^2^X, R^2^Y, and Q^2^, where Q^2^ indicates the predictive power of the model, and R^2^X and R^2^Y indicate the rate of explanation of the X and Y matrices, respectively, by the constructed model. The closer these three indicators are to 1, the more stable and reliable the model. A valid model is indicated by Q^2^ > 0.5. In this study, all of the OPLS-DA models exhibited R^2^X values greater than 0.7, and both R^2^Y and Q^2^ values were greater than 0.9, indicating outstanding performance in explaining independent and dependent variables, as well as for predicting new data. On the basis of the permutation tests, these models were not overfitted.

To obtain more valuable information, a *t*-test was also implemented with SPSS 25.0 (IBM, USA). In the end, the DAMs were screened according to the following thresholds: VIP > 1, *p*-value of *t*-test < 0.05, and Log_2_ FC > 0 or <0. The DAMS were visualized in volcano plots generated using SRpolt (https://www.bioinformatics.com.cn, accessed on 28 September 2024).

#### 4.6.3. Metabolic Pathway Analysis

The DAMs were annotated using the KEGG database. An enrichment analysis to determine the biosynthetic and metabolic pathways associated with the differential components was conducted using MetaboAnalyst 6.0 (https://www.metaboanalyst.ca/, accessed on 11 October 2024). The results are shown as bubble diagrams. The biosynthetic pathways of phenolic acids and coumarins, which are the main secondary metabolites of NI, were constructed based on information reported in the literature. Clustered heat maps were constructed to display the distribution of secondary metabolites related to the pathway maps in the nine batches of samples. These revealed the key metabolic components responsible for the metabolic differences between the wild and cultivated NI from different origins. Pearson’s correlation analysis was performed using Origin 2022 software (OriginLab, Northampton, MA, USA). The correlation analyses were conducted to detect relationships between the amino acid components of wild NI and NI cultivated in Gansu and Qinghai and the phenolic acid and coumarin components of the above metabolic pathways. These analyses revealed the potential relationships between endogenous substances in NI and its secondary metabolism. The correlation heatmaps were plotted using the Correlation Plot Application in Origin 2022 software, with blue ovals indicating negative correlations, red ovals indicating positive correlations, and narrower ovals indicating larger correlation coefficients. Asterisks indicate significant correlations between two components, where * and ** indicate significance at the *p* < 0.05 level and the *p* < 0.01 level, respectively.

#### 4.6.4. Statistical Analyses of the Results of Pharmacological Experiments

The results of the pharmacological experiments are expressed as mean ± SE (standard error). The number of neutrophils and gene transcript levels was compared between the model group and the control and experimental groups separately via a *t*-test, using SPSS 25.0 (IBM, Armonk, NY, USA). Differences were considered significant at *p* < 0.05. In the figures, asterisks indicate significant differences between the model group and the control/treatment groups, with * and ** indicating significant differences at *p* < 0.05 and *p* < 0.01, respectively. Histograms were generated using GraphPad Prism 9.5.0 (GraphPad Software, La Jolla, CA, USA).

## 5. Conclusions

In this study, wild and cultivated NI from Sichuan, Qinghai, and Gansu were subjected to metabolic analyses. The results showed that the chemical compositions of NI differed, depending on the origin, as well as between wild and cultivated materials. The total contents of angular coumarins, linear coumarins, and simple coumarins differed between wild NI and cultivated NI from Gansu and Qinghai, while the contents of the vast majority of phytometabolites, such as angular coumarins and linear coumarins, in cultivated NI from Sichuan are significantly lower than those in other candidates. Notably, the contents of angelicin (C77), 6′-*O*-β-D-glucoxyl-7′-hydroxyberganottin (C80), and 8-geranyl-5-methoxy-psoralen (C107) are significantly higher in cultivated NI from Sichuan than in the other candidates. The differential metabolic pathways between wild and cultivated NI inlcuded arginine biosynthesis; alanine, aspartic acid, and glutamic acid metabolism; arginine and proline metabolism; and phenylalanine metabolism. The differential metabolic pathways between cultivated NI from Sichuan and cultivated NI from other origins included phenylalanine, tyrosine, and tryptophan metabolism, as well as arginine biosynthesis. Analyses of these biosynthetic pathways revealed seven key metabolic intermediates in the biosynthesis of phenolic acids and coumarins that were key factors contributing to the differences in the compositions of phenolic acid and coumarin components formed by NI’ s origins and growth modes, i.e., cinnamic acid, *p*-coumaric acid, *p*-coumaroyl quinic acid, umbelliferone, osthenol, demethylsuberosin, and aesculetin. We detected significant correlations between amino acids and coumarins and between some amino acids and phenolic acid biosynthesis. A pharmacodynamic study using the yolk sac model in transgenic neutrophil green fluorescent zebrafish (MPX) showed that both wild and cultivated NI from Sichuan exhibited anti-inflammatory pharmacological effects. The results of this study provide a basis for further research on NI resources and their cultivation, as well as for the development and application of products from these materials.

## Figures and Tables

**Table 1:** Information for nine batches of *N. incisum* samples.

Serial Number	Sample Name	Code Name	Harvest Site	Harvest Time
1	Sichuan wild product	SW	Aba, Sichuan	2023.11
2	Qinghai wild product	HW	Tanggula Mountains	2023.11
3	Gansu wild product	GW	Min County, Gansu	2023.12
4	Sichuan cultivated product (4-year-old)	SC-1	Aba, Sichuan	2023.11
5	Sichuan cultivated product (4-year-old)	SC-2	Aba, Sichuan	2023.11
6	Qinghai cultivated product (4-year-old)	HC-1	Haixi Mongol and Tibet Autonomous Prefecture	2023.11
7	Qinghai cultivated product (4-year-old)	HC-2	Haixi Mongol and Tibet Autonomous Prefecture	2023.11
8	Gansu cultivated product (4-year-old)	GC-1	Longnan, Gansu	2023.12
9	Gansu cultivated product (4-year-old)	GC-2	Longnan, Gansu	2023.12

## Data Availability

All data relevant to this study are contained within the article or Supplementary Materials.

## Supplementary Materials

The following supporting information can be downloaded at: https://www.mdpi.com/article/10.3390/molecules30030468/s1, Figure S1: Structures of all identified components. Table S1: Volatile components information. Table S2: Non-volatile components information. Table S3: Results of neutrophil counts (*n* = 10). Table S4: Results of gene expressions (*n* = 3). Table S5: Information on reference standards for compound identification. Table S6: Results of maximum detectable concentration determination (*n* = 30). Table S7: Concentrations of total RNA and A260/A280 ratio (*n* = 3). Table S8: Primer sequences for q-PCR. Refs [40,41,42,43,44,45,46,47,48,49,50,51,52,53,54,55,56,57,58,59,60,61,62,63,64,65,66,67,68,69,70,71,72,73] are cited in Supplementary Materials.
