# Supplementary material for: Comparative Analysis of Metabolites of Wild and Cultivated Notopterygium incisum from Different Origins and Evaluation of Their Anti-Inflammatory Activity
Source: Molecules. 2025 Jan 22;30(3):468. doi: 10.3390/molecules30030468 (PMC11820002; doi:10.3390/molecules30030468)
Supplement: Supplementary file 1 [file molecules-30-00468-s001.zip › Figure S1.pdf]

Simple coumarins：

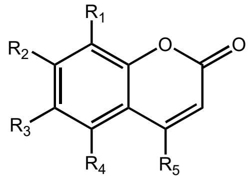

C36 R_1= H, R_2= OH, R_3= O-Glc, R_4= H, R_5= H
C40 R_1= H, R_2= OH, R_3= OH, R_4= H, R_5= H
C42 R_1= O-Glc, R_2= OH, R_3= OMe, R_4= H, R_5= H
C45 R_1= OH, R_2= OH, R_3= OMe, R_4= H, R_5= H
C46 R_1= H, R_2= O-Glc, R_3= OMe, R_4= H, R_5= H
C50 R_1= H, R_2= OH, R_3= H, R_4= H, R_5= H
C51 R_1= H, R_2= OH, R_3= OMe, R_4= H, R_5= H
C56 R_1= OMe, R_2= OH, R_3= OMe, R_4= H, R_5= H
C63 R_1= H, R_2= O-Glc, R_3= H, R_4= H, R_5= CH_3
C67 R_1= H, R_2= OMe, R_3= OH, R_4= H, R_5= H
C87 R_1= C_5H_9, R_2= OH, R_3= H, R_4= H, R_5= H
C89 R_1= H, R_2= O-C_5H_9, R_3= OMe, R_4= H, R_5= H
C90 R_1= H, R_2= OMe, R_3= H, R_4= O-C_5H_9, R_5= H
C91 R_1= H, R_2= OH, R_3= C_5H_9, R_4= H, R_5= H
C96 R_1= H, R_2= O-C_5H_9, R_3= H, R_4= H, R_5= H
C100 R_1= H, R_2= O-$C_5H_8CH(OH)C_4H_7$, R_3= H, R_4= H, R_5= H
C103 R_1= H, R_2= OH, R_3= $C_{10}H_{17}$, R_4= H, R_5= H
C105 R_1= H, R_2= O-$C_{10}H_{17}$, R_3= OMe, R_4= H, R_5= H
C108 R_1= H, R_2= O-$C_{10}H_{17}$, R_3= H, R_4= H, R_5= H

Linear furanocoumarins：

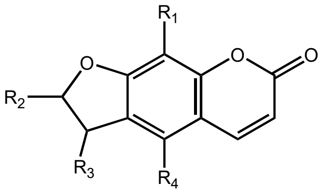

C48 R_1= H, R_2= C_3H_6-O-Glc, R_3= OH, R_4= H
C55 R_1= H, R_2= H, R_3= H, R_4= O-Glc
C59 R_1= H, R_2= C_3H_6-O-Glc, R_3= H, R_4= H
C61 R_1= H, R_2= C_3H_6-O-Glc-Glc, R_3= H, R_4= H
C62 R_1= H, R_2= C_3H_6-O-Glc, R_3= H, R_4= H
C66 R_1= OH, R_2= H, R_3= H, R_4= H
C69 R_1= H, R_2= O-$CH_2CH(OH)C_3H_6OH$, R_3= H, R_4= H
C70 R_1= H, R_2= C_3H_6OH, R_3= H, R_4= H
C72 R_1= H, R_2= H, R_3= H, R_4= OH
C74 R_1= H, R_2= H, R_3= H, R_4= O-$CH_2CH(OH)C_3H_6OH$
C75 R_1= H, R_2= O-$CH_2CH(OH)C_3H_6OH$, R_3= H, R_4= OMe
C76 R_1= H, R_2= C_3H_6OH, R_3= H, R_4= H
C78 R_1= H, R_2= H, R_3= H, R_4= H
C79 R_1= H, R_2= C_3H_6-O-Glc-O-$C_{10}H_9O_3$, R_3= H, R_4= H
C80 R_1= H, R_2= H, R_3= H, R_4= O-$C_6H_{10}CH$(O-Glc)C_3H_6OH
C81 R_1= H, R_2= H, R_3= H, R_4= OMe
C83 R_1= H, R_2= H, R_3= H, R_4= O-$CH_2CH(OH)C_3H_5$
C84 R_1= OH, R_2= H, R_3= H, R_4= OMe
C88 R_1= H, R_2= H, R_3= H, R_4= O-$C_2H_3OC_3H_6$
C94 R_1= C_5H_9, R_2= H, R_3= H, R_4= OH
C95 R_1= O-C_5H_9, R_2= H, R_3= H, R_4= H
C97 R_1= H, R_2= H, R_3= H, R_4= O-$C_5H_8CH(OH)C_4H_7$
C99 R_1= O-C_5H_9, R_2= H, R_3= H, R_4= OMe
C101 R_1= H, R_2= H, R_3= H, R_4= O-C_5H_9
C107 R_1= O-$C_{10}H_{17}$, R_2= H, R_3= H, R_4= OMe

Angular furanocoumarins：

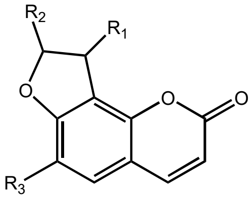

C52 R_1= OH, R_2= C_3H_6-O-Glc, R_3= H
C57 R_1= H, R_2= C_3H_6-O-Glc-Glc, R_3= H
C58 R_1= H, R_2= C_3H_6-O-Glc, R_3= H
C60 R_1= OH, R_2= C_3H_6OH, R_3= H
C73 R_1= H, R_2= C_3H_6OH, R_3= H
C77 R_1= H, R_2= H, R_3= H
C85 R_1= H, R_2= H, R_3= OMe

Amino acids：

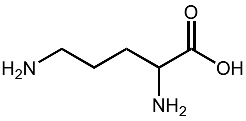
C1

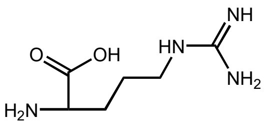
C2

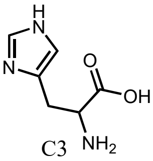
C3

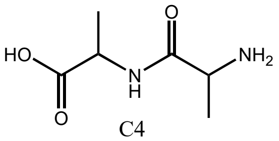
C4

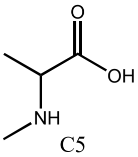
C5

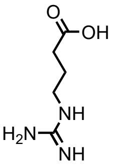
C8

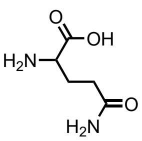
C10

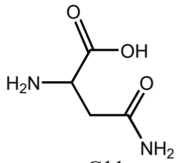
C11

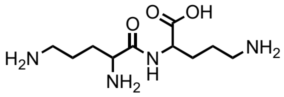
C12

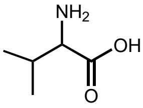
C14

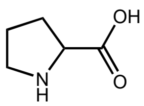
C16

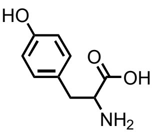
C25

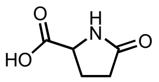
C30

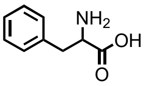
C33

Flavonoids：

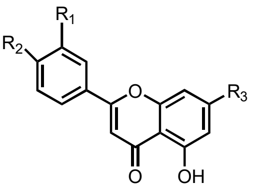

C65 R_1= OH, R_2= OMe, R_3= O-Glc-Rha
C82 R_1= OH, R_2= OMe, R_3= OH

Nucleosides:

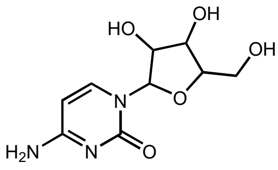

C6

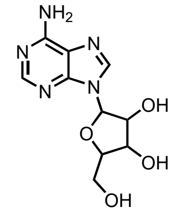

C21

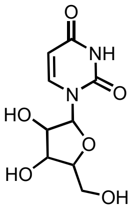

C24

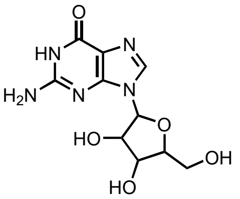

C26

Polyene-alkynes:

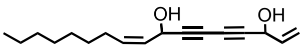

C102

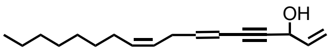

C104

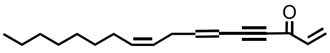

C110

Carbohydrates :

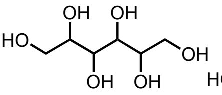

C15

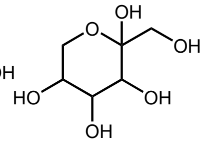

C17

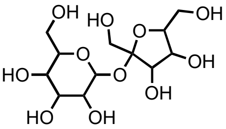

C18

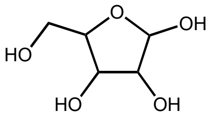

C19

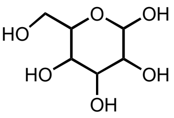

C29

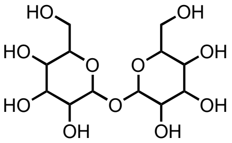

C31

Phenolic acids and derivatives:

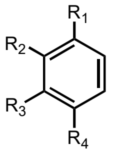

C34 R_1= COOH, R_2= H, R_3= OMe, R_4= OH
C35 R_1= COOH, R_2= H, R_3= OH, R_4= OH
C37 R_1= C_2H_2COOH, R_2= H, R_3= H, R_4= H
C38 R_1= COOH, R_2= OH, R_3= H, R_4= H
C39 R_1= $C_2H_2COOC_6H_7(OH)_3COOH$, R_2= H, R_3= OH, R_4= OH
C41 R_1= C_2H_2COOH, R_2= H, R_3= OH, R_4= OH
C43 R_1= C_2H_2COOH, R_2= H, R_3= H, R_4= OMe
C44 R_1= $C_2H_2COOC_6H_7(OH)_3COOH$, R_2= H, R_3= H, R_4= OH
C47 R_1= C_2H_2COOH, R_2= H, R_3= H, R_4= OH
C49 R_1= $C_2H_2COOC_6H_7(OH)_3COOH$, R_2= H, R_3= OMe, R_4= OH
C53 R_1= C_2H_2COOH, R_2= H, R_3= OMe, R_4= OH
C54 R_1= $C_2H_2COOC_6H_7(OH)_3COOH$, R_2= H, R_3= OMe, R_4= OH
C64 R_1= $C_2H_2COOC_{16}H_{17}O_8$, R_2= H, R_3= OH, R_4= OH
C71 R_1= C_2H_2COOH, R_2= H, R_3= OMe, R_4= OMe
C86 R_1= $C_2H_2COOC_2H_4C_6H_4OH$, R_2= H, R_3= OMe, R_4= OH
C92 R_1= $COOC_2H_4C_6H_4OH$, R_2= H, R_3= H, R_4= OMe
C93 R_1= $COOC_2H_4C_6H_4OMe$, R_2= H, R_3= OMe, R_4= OH
C98 R_1= $C_2H_2COOC_2H_4C_6H_5$, R_2= H, R_3= OMe, R_4= OH
C106 R_1= $C_2H_2COOC_{10}H_{17}$, R_2= H, R_3= OMe, R_4= OH

Fatty acid:

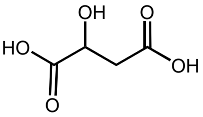

C22

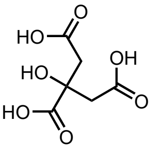

C23

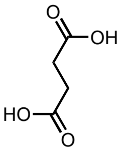

C27

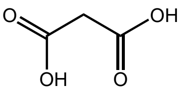

C28

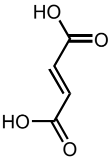

C32

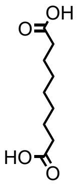

C68

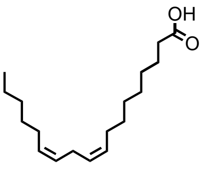

C112

Amides:

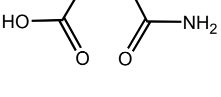

C9

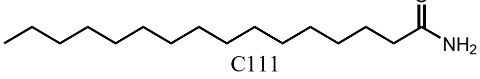

C111

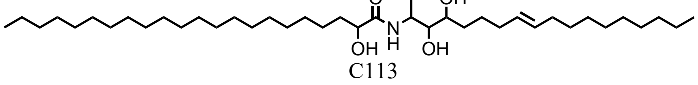

C113

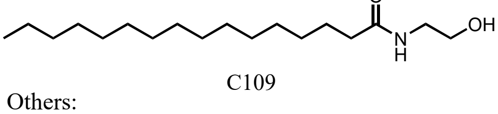

C109

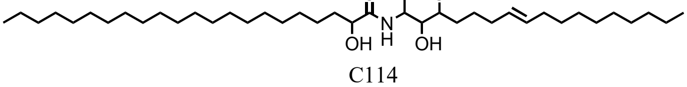

C114

Others:

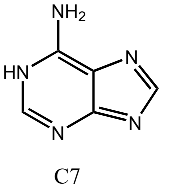

C7

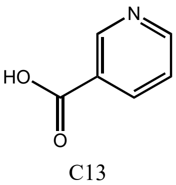

C13

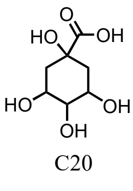

C20
