# Supplementary material for: Comparative Analysis of Metabolites of Wild and Cultivated Notopterygium incisum from Different Origins and Evaluation of Their Anti-Inflammatory Activity
Source: Molecules. 2025 Jan 22;30(3):468. doi: 10.3390/molecules30030468 (PMC11820002; doi:10.3390/molecules30030468)
Supplement: Supplementary file 1 [file molecules-30-00468-s001.zip › Reference for comound identification.pdf]

Table S2 Ref [40-73].

40. Wu, M.R.; Tang, L.H.; Chen, Y.Y.; Shu, L.X.; Xu, Y.Y.; Yao, Y.Q.; Li, Y.B. Systematic characterization of the chemical constituents in vitro and in vivo of Qianghuo by UPLC-Q-TOF-MS/MS. *Fitoterapia.* **2024**, *172,* 105758. [CrossRef]
41. Liu, Y.M.; Wang, S.; Dickenson, A.; Mao, J.; Bai, X.; Liao, X. An on-line SPE-LC-MS/MS method for quantification of nucleobases and nucleosides present in biological fluids. *Anal Methods.* **2024**, *16,* 2505-2512. [CrossRef]
42. Yu, Y.; Yao, Q.; Chen, D.; Zhang, Z.; Pan, Q.; Yu, J.; Cao, H.; Li, L.; Li, L. Serum metabonomics reveal the effectiveness of human placental mesenchymal stem cell therapy for primary sclerosing cholangitis. *Stem Cell Res Ther.* **2024**, *15,* 346. [CrossRef]
43. Ndolo, V.U.; Fulcher, R.G.; Beta, T. Application of LC–MS–MS to identify niacin in aleurone layers of yellow corn, barley and wheat kernels. *J. Cereal Sci.* **2015**, *65,* 88-95. [CrossRef]
44. Shevchuk, A.; Megias-Perez, R.; Zemedie, Y.; Kuhnert, N. Evaluation of carbohydrates and quality parameters in six types of commercial teas by targeted statistical analysis. *Food Res Int.* **2020**, *133,* 109122. [CrossRef]
45. Choi, J.N.; Kim, J.; Ponnusamy, K.; Lim, C.; Kim, J.G.; Muthaiya, M.J.; Lee, C. Metabolic changes of Phomopsis longicolla fermentation and its effect on antimicrobial activity against Xanthomonas oryzae. *J Microbiol Biotechnol.* **2013**, *23,* 177-183. [CrossRef]
46. Zhang, P.; Yang, X.W. Studies on chemical constituents in roots and rhizomes of Notopterygium incisum. *China J Chin Mater Med.* **2008**, *33,* 2918-2921. [PubMed]
47. Ma, X.; Wu, Y.; Li, Y.; Huang, Y.; Liu, Y.; Luo, P.; Zhang, Z. Rapid discrimination of Notopterygium incisum and Notopterygium franchetii based on characteristic compound profiles detected by UHPLC - QTOF - MS/MS coupled with multivariate analysis. *Phytochem Anal.* **2020**, *31,* 355-365. [CrossRef]
48. Fontal, M.; van Drooge, B.L.; Lopez, J.F.; Fernandez, P.; Grimalt, J.O. Broad spectrum analysis of polar and apolar organic compounds in submicron atmospheric particles. *J Chromatogr A.* **2015**, *1404,* 28-38. [CrossRef]
49. Duan, H.; Wang, W.; Li, Y.; Jilany Khan, G.; Chen, Y.; Shen, T.; Bao, N.; Hua, J.; Xue, Z.; Zhai, K.; Wei, Z. Identification of phytochemicals and antioxidant activity of Premna microphylla Turcz. stem through UPLC-LTQ-Orbitrap-MS. *Food Chem.* **2022**, *373,* 131482. [CrossRef]
50. Tian, Y.J.; Li, J.N.; Feng, J.L.; Wang, Q.; Zhang, C.Y.; Zhong, H.G.; Cheng, W.Q.; Yang, C. Chemical constituents from Notopterygium Root. *Liaoning J Tradit Chin Med.* **2013**, *15,* 40-42. DOI: 10.13194/j.jlunivtcm.2013.06.42.tianyj.078. [CrossRef]
51. Li, L.M.; Liang, B.D.; Yu, S.W.; Sun, H.D. Chemical constituents of Notopterygium incisum. *Chin J Nat Med.* **2007**, *05*, 351-354.
52. Dadabay, C.Y.; Spaulding, P.B.; Valenzuela, E.; Turner, M.; Eckert, K.E.; Julkunen-Tiitto, R.; Noblit, N.; Mansfield, D.H. Polyphenols from the sagebrush Artemisia tridentata ssp. tridentata affect the redox state of cultured hepatocytes by direct and indirect mechanisms. *Curr Top Phytochem.* **2019**, *15,* 15-25. [PubMed]
53. Cao, L.; Zhang, S.; Cao, J.; Chang, R.; Qu, C.; Li, C.; Yan, J.; Quan, X.; Xu, Z.; Liu, G. Nitrogen modifies wood composition in poplar seedlings by regulating carbon and

nitrogen metabolism. *Industrial Crops and Products.* **2024**, *219,* 119118.
54. Yang, J.; Fu, C.W.; Qin, H.L.; Qin, D.J.; Qin, Z.L. Geographical origin traceability of Desmodium caudatum (Thunb.) DC. By UPLC MS/MS coupled with BP neural network. *Chin J Pharm Anal.* **2024**, *44,* 1176-1185. [CrossRef]
55. Su, X.; Wu, Y.; Li, Y.; Huang, Y.; Liu, Y.; Luo, P.; Zhang, Z. Effect of Different Post-Harvest Processing Methods on the Chemical Constituents of Notopterygium franchetii by an UHPLC-QTOF-MS-MS Metabolomics Approach. *Molecules.* **2019**, *24,* 3188. [CrossRef]
56. You, M.; Xiong, J.; Zhao, Y.; Cao, L.; Wu, S.B.; Xia, G.; Hu, J.F. Glycosides from the methanol extract of Notopterygium incisum. *Planta Med.* **2011**, *77,* 1939-1943. [CrossRef]
57. Wu, X.W.; Zhang, Y.B.; Zhang, L.; Yang, X.W. Simultaneous quantification of 33 active components in Notopterygii Rhizoma et Radix using ultra high performance liquid chromatography with tandem mass spectrometry. *J Chromatogr B Analyt Technol Biomed Life Sci.* **2018**, *1092,* 244-251. [CrossRef]
58. Xu, K.; Jiang, S.; Zhou, Y.; Zhang, Y.; Xia, B.; Xu, X.; Zhou, Y.; Li, Y.; Wang, M.; Ding, L. Discrimination of the seeds of Notopterygium incisum and Notopterygium franchetii by validated HPLC-DAD-ESI-MS method and principal component analysis. *J Pharm Biomed Anal.* **2011**, *56,* 1089-1093. [CrossRef]
59. González-Trujano, M.; Ventura-Martínez, R.; Chávez, M.; Díaz-Reval, I.; Pellicer, F. Spasmolytic and Antinociceptive Activities of Ursolic Acid and Acacetin Identified in Agastache mexicana. *Planta Med.* **2012**, *78,* 793-796. [CrossRef]
60. Wu, S.B.; Zhao, Y.; Fan, H.; Hu, Y.H.; Hamann, M.T.; Peng, J.N.; Starks, C.M.; O'Neil-Johnson, M.; Hu, J.F. New guaiane sesquiterpenes and furanocoumarins from Notopterygium incisum. *Planta Med.* **2008**, *74,* 1812-1817. [CrossRef]
61. Ding, X.-F.; Feng, X.; Dong, Y.-F.; Zhao, X.-Z.; Chen, Y.; Wang, M. Studies on chemical constituents of the roots of Angelica pubescens. *China J Chin Mater Med.* **2008**, *31,* 516-518.
62. Matano, Y.; Okuyama, T.; Shibata, S.; Hoson, M.; Kawada, T.; Osada, H.; Noguchi, T. Studies on Coumarins of a Chinese Drug "Qian-Hu"; VII1. Structures of New Coumarin-Glycosides of Zi-Huan Quian-Hu and Effect of Coumarin-Glycosides on Human Platelet Aggregation. *Planta Med.* **1986**, *52,* 135-138. [CrossRef]
63. Xiao, L.; Zhou, Y.M.; Zhang, X.F.; Du, F.Y. Notopterygium incisum extract and associated secondary metabolites inhibit apple fruit fungal pathogens. *Pestic Biochem Physiol.* **2018**, *150,* 59-65. [CrossRef]
64. Ma, Z.; Xu, W.; Liu-Chen, L.Y.; Lee, D.Y. Novel coumarin glycoside and phenethyl vanillate from Notopterygium forbesii and their binding affinities for opioid and dopamine receptors. *Bioorg Med Chem.* **2008**, *16,* 3218-3223. [CrossRef]
65. Ma, B.; Liu, X.; Lu, Y.; Ma, X.; Wu, X.; Wang, X.; Jia, M.; Su, P.; Tong, Y.; Guan, H.; Jiang, Z.; Gao, J.; Huang, L.; Gao, W. A specific UDP-glucosyltransferase catalyzes the formation of triptophenolide glucoside from Tripterygium wilfordii Hook. f. *Phytochemistry.* **2019**, *166,* 112062. [CrossRef]
66. Deng, G.-G.; Yang, X.-W.; Zhang, Y.-B.; Xu, W.; Wei, W.; Chen, T.-L. [Chemical constituents from lipophilic parts in roots of Angelica dahurica var. formosana cv. Chuanbaizhi]. *China J Chin Mater Med.* **2015**, *40,* 2148-2156.
67. Hu, Z.; Chen, J.-T.; Jiang, S.-C.; Liu, Z.; Ge, S.-B.; Zhang, Z. Chemical components and functions of Taxus chinensis extract. *JKSU-Science.* **2020**, *32,* 1562-1568. [CrossRef]

68. Zhang, Y.; Bai, P.; Zhuang, Y.; Liu, T. Two O-Methyltransferases Mediate Multiple Methylation Steps in the Biosynthesis of Coumarins in Cnidium monnieri. *J Nat Prod.* **2022**, *85,* 2116-2121. [CrossRef]
69. Ayoub, N.; Al - Azizi, M.; König, W.; Kubeczka, K.H. Essential oils and a novel polyacetylene from Eryngium yuccifolium Michaux. (Apiaceae). *Flavour Frag J.* **2006**, *21,* 864-868. [CrossRef]
70. Bindila, L.; Lutz, B. Extraction and Simultaneous Quantification of Endocannabinoids and Endocannabinoid-Like Lipids in Biological Tissues. *Methods Mol Biol.* **2016**, *1412,* 9-18. [CrossRef]
71 Tsochatzis, E.; Lopes, J.; Gika, H.; Theodoridis, G. Polystyrene Biodegradation by Tenebrio molitor Larvae: Identification of Generated Substances Using a GC-MS Untargeted Screening Method. *Polymers.* **2020**, *13,* 17. [CrossRef]
72. Zhang, S.; You, J.; Zhou, G.; Li, C.; Suo, Y. Analysis of free fatty acids in Notopterygium forbesii Boiss by a novel HPLC method with fluorescence detection. *Talanta.* **2012**, *98,* 95-100. [CrossRef]
73. Lin, W.Y.; Yen, M.H.; Teng, C.M.; Tsai, I.L.; Chen, I.S. Cerebrosides from the Rhizomes of Gynura Japonica. *J Chin Chem Soc-Tai.* **2004**, *51,* 1429-1434.
