# Supplementary material for: Comparative Analysis of Metabolites of Wild and Cultivated Notopterygium incisum from Different Origins and Evaluation of Their Anti-Inflammatory Activity
Source: Molecules. 2025 Jan 22;30(3):468. doi: 10.3390/molecules30030468 (PMC11820002; doi:10.3390/molecules30030468)
Supplement: Supplementary file 1 [file molecules-30-00468-s001.zip › Supplementary Material Table S3-S8.pdf]

Table S3. Results of neutrophil counts (n = 10).

Group	Concentration (μg/mL)	Number of neutrophils (number, mean ± SE)
**Control**	-	13.1 ± 0.912
**Model**	-	24.2 ± 1.69
**Dexamethasone acetate**	43.5	15.3 ± 1.19
	0.977	20.0 ± 1.15
**SW**	1.95	19.4 ± 0.819
	3.91	17.7 ± 1.04
	0.977	22.7 ± 0.716
**SC-2**	1.95	18.1 ± 1.28
	3.91	15.8 ± 1.91

Table S4. Results of gene expressions (n = 3).

Group	Concentration (μg/mL)	*IL-1β* relative expression (mean ± SE)	*IL-6* relative expression (mean ± SE)	*TNF-α* relative expression (mean ± SE)
**Control**	-	0.209 ± 0.029	0.278 ± 0.099	0.300 ± 0.026
**Model**	-	1.00 ± 0.109	1.00 ± 0.075	1.00 ± 0.182
**Dexamethasone acetate**	43.5	0.299 ± 0.015	0.369 ± 0.089	0.423 ± 0.094
**SW**	0.977	0.848 ± 0.084	0.507 ± 0.032	0.891 ± 0.295
	1.95	0.701 ± 0.095	0.290 ± 0.147	0.933 ± 0.203
	3.91	0.504 ± 0.025	0.698 ± 0.211	1.63 ± 0.160
**SC-2**	0.977	0.697 ± 0.068	0.635 ± 0.092	0.704 ± 0.074
	1.95	0.669 ± 0.069	0.284 ± 0.044	1.24 ± 0.098
	3.91	0.357 ± 0.078	1.15 ± 0.195	1.45 ± 0.078

Table S5. Information of reference standards for compound identification.

Name	Batch number	Provider	Supplier City	Country
p-Coumaric acid	RDD-D03211712016	Rifenside Biotechnology Co., Ltd	Chengdu	China
Falcarinol	RDD-F02502408030			
Bergaptol	RDD-F02611811008			
Phellopterin	RDD-S24702408030			
Phenethyl ferulate	RDD-A01802307027			
Notopterol	RFS-Q01002111011			
Isoimperatorin	RFS-Y00711704026			

Imperatorin	O-001-180525			
Caffeic acid	RFS-K00311812016			
Oxypeucedanin hydrate	RFS-S12811804026			
Nodakenin	RFS-Z01001905014			
Scopoletin	D-020-190513			
Isobergapten	Y-127-180327			
Xanthotoxol	H-065-181216			
Psoralen	000051-202308	Jiangxi Baicaoyuan Biotechnology Co., Ltd.	Nanchang	China
Umbelliferone	000690-202309			
Columbianetin	000328-202312			
Chlorogenic acid	000315-202401			
Aesculetin	000614-202305			
Isochlorogenic acid B	000319-202012			
Angelicin	18033005	Chengdu Pufide Biotechnology Co., Ltd	Chengdu	China
Fraxin	17072707			
Ferulic acid	5870	China National Institute for Food and Drug Control	Beijing	China

Following the ″experimental conditions of 4.5.1″, the maximum detectable concentration (MTC) of anti-bacterial inflammatory efficacy of Sichuan wild (SW) product was 3.91 μg/mL and 7.81 μg/mL for the Sichuan cultivated product (SC-2). See Table S6 for details.

Table S6. Results of maximum detectable concentration determination (n = 30).

Groups	Concentration（μg/mL）	Deaths	Mortality rate（%）	Phenotype
**Normal control group**	-	0	0	No apparent abnormality
**Model control group**	-	0	0	No apparent abnormality
**SW**	0.977	0	0	Similar status to model
	1.95	0	0	Similar status to model
	3.91	0	0	Similar status to model
	7.81	0	0	Poorer status than model
	15.6	0	0	Poorer status than model
**SC-2**	0.977	0	0	Similar status to model
	1.95	0	0	Similar status to model
	3.91	0	0	Similar status to model
	7.81	0	0	Similar status to model
	15.6	0	0	Poorer status than model

After sample processing, total RNA of zebrafish was extracted, and the concentration of RNA and the A260/A280 ratio were determined by UV-visible spectrophotometer (Table S7). The A260/A280 ratios were between 1.8 and 2.2, indicating that the total RNA of zebrafish obtained by the extraction was of better quality, and it could be used for the subsequent q-PCR experiments.

The primer sequences are shown in Table S8.

Table S7. Concentrations of total RNA and A260/A280 ratios (n = 3).

Group	Concentration	RNA concentration（μg/μL）			A260/A280		
	（μg/mL）	Sample 1	Sample 2	Sample 3	Sample 1	Sample 2	Sample 3
Control	-	0.695	0.701	0.702	1.96	1.97	1.98
Model	-	0.590	0.740	0.742	1.94	1.95	1.97
Dexamethasone acetate	43.5	0.750	0.545	0.544	1.97	1.97	1.97
SW	0.977	0.598	0.776	0.767	1.94	1.96	1.97
	1.95	0.558	0.577	0.582	1.96	1.95	1.96
	3.91	0.587	0.737	0.730	1.94	1.94	1.94
SC-2	0.977	0.685	0.679	0.678	1.92	1.94	1.92
	1.95	0.736	0.707	0.703	1.97	1.98	1.97
	3.91	0.737	0.804	0.795	1.97	1.96	1.97

Table S8. Primer sequences for q-PCR.

Genes	Primer Sequences	
**β-actin**	Forward	5′-TCGAGCAGGAGATGGGAACC-3′
	Reverse	5′-CTCGTGGATACCGCAAGATTC-3′
**IL-6**	Forward	5′-TCAACTTCTCCAGCGTGATG-3′
	Reverse	5′-TCTTTCCCTCTTTTCCTCCTG-3′
**IL-1β**	Forward	5′-GAACAGAATGAAGCACATCAAACC-3′
	Reverse	5′-ACGGCACTGAATCCACCAC-3′
**TNF-α**	Forward	5′-GCGCTTTTCTGAATCCTACG-3′
	Reverse	5′-TGCCCAGTCTGTCTCCTTCT-3′
